# Lessons for the clinical nephrologist: recurrence of nephrotic syndrome induced by SARS-CoV-2

**DOI:** 10.1007/s40620-020-00855-5

**Published:** 2020-09-05

**Authors:** Adrian A. N. Doevelaar, Bodo Hölzer, Felix S. Seibert, Frederic Bauer, Ulrik Stervbo, Benjamin J. Rohn, Panagiota Zgoura, Peter Schenker, Eva Vonbrunn, Kerstin Amann, Richard Viebahn, Nina Babel, Timm H. Westhoff

**Affiliations:** 1 Medical Department 1, University Hospital Marien Hospital Herne, Ruhr-University Bochum, Hölkeskampring 40, 44625 Herne, Germany; 2 Center for Translational Medicine, University Hospital Marien Hospital Herne, Ruhr-University Bochum, Herne, Germany; 3 Department of Surgery, University Hospital Knappschaftskrankenhaus Bochum, Ruhr-University Bochum, Bochum, Germany; 4 Department of Nephropathology, Friedrich-Alexander University (FAU) Erlangen-Nürnberg, Erlangen, Germany

**Keywords:** SARS-CoV-2, COVID-19, Podocytopathy

## Abstract

SARS-CoV-2 is characterized by a multiorgan tropism including the kidneys. Recent autopsy series indicated that SARS-CoV-2 can infect both tubular and glomerular cells. Whereas tubular cell infiltration may contribute to acute kidney injury, data on a potential clinical correlative to glomerular affection is rare. We describe the first case of nephrotic syndrome in the context of COVID-19 in a renal transplant recipient. A 35 year old male patient received a kidney allograft for primary focal segmental glomerulosclerosis (FSGS). Three months posttransplant a recurrence of podocytopathy was successfully managed by plasma exchange, ivIG, and a conversion from tacrolimus to belatacept (initial proteinuria > 6 g/l decreased to 169 mg/l). Six weeks later he was tested positive for SARS-CoV-2 and developed a second increase of proteinuria (5.6 g/l). Renal allograft biopsy revealed diffuse podocyte effacement and was positive for SARS-CoV-2 in RNA in-situ hybridation indicating a SARS-CoV-2 associated recurrence of podocytopathy. Noteworthy, nephrotic proteinuria resolved spontaneously after recovering from COVID-19. The present case expands the spectrum of renal involvement in COVID-19 from acute tubular injury to podocytopathy in renal transplant recipients. Thus, it may be wise to test for SARS-CoV-2 prior to initiation of immunosuppression in new onset glomerulopathy during the pandemic.

**Electronic supplementary material:**

The online version of this article (10.1007/s40620-020-00855-5) contains supplementary material, which is available to authorized users.

## Case

A 35-year old male patient of African origin with primary focal segmental glomerulosclerosis (FSGS) underwent successful renal transplantation in December 2019. After induction therapy with thymoglobuline, immunosuppression consisted of tacrolimus, mycophenolate mofetil, and prednisolone. 3 weeks after transplantation FSGS recurred with an increase of proteinuria to > 7 g/l (> 7 g/g creatinine). Electron microscopy showed diffuse podocyte effacement consistent with recurrence of the underlying disease. This glomerulopathy was successfully treated by five sessions of plasmapheresis, ivIG (3 × 0.5 g/kg bodyweight), and conversion from tacrolimus to belatacept. The remission of FSGS remained stable until recurrence of nephrotic proteinuria (3286 mg/l) was detected in a routine visit 16 weeks after transplantation.

**Fig. 1:**
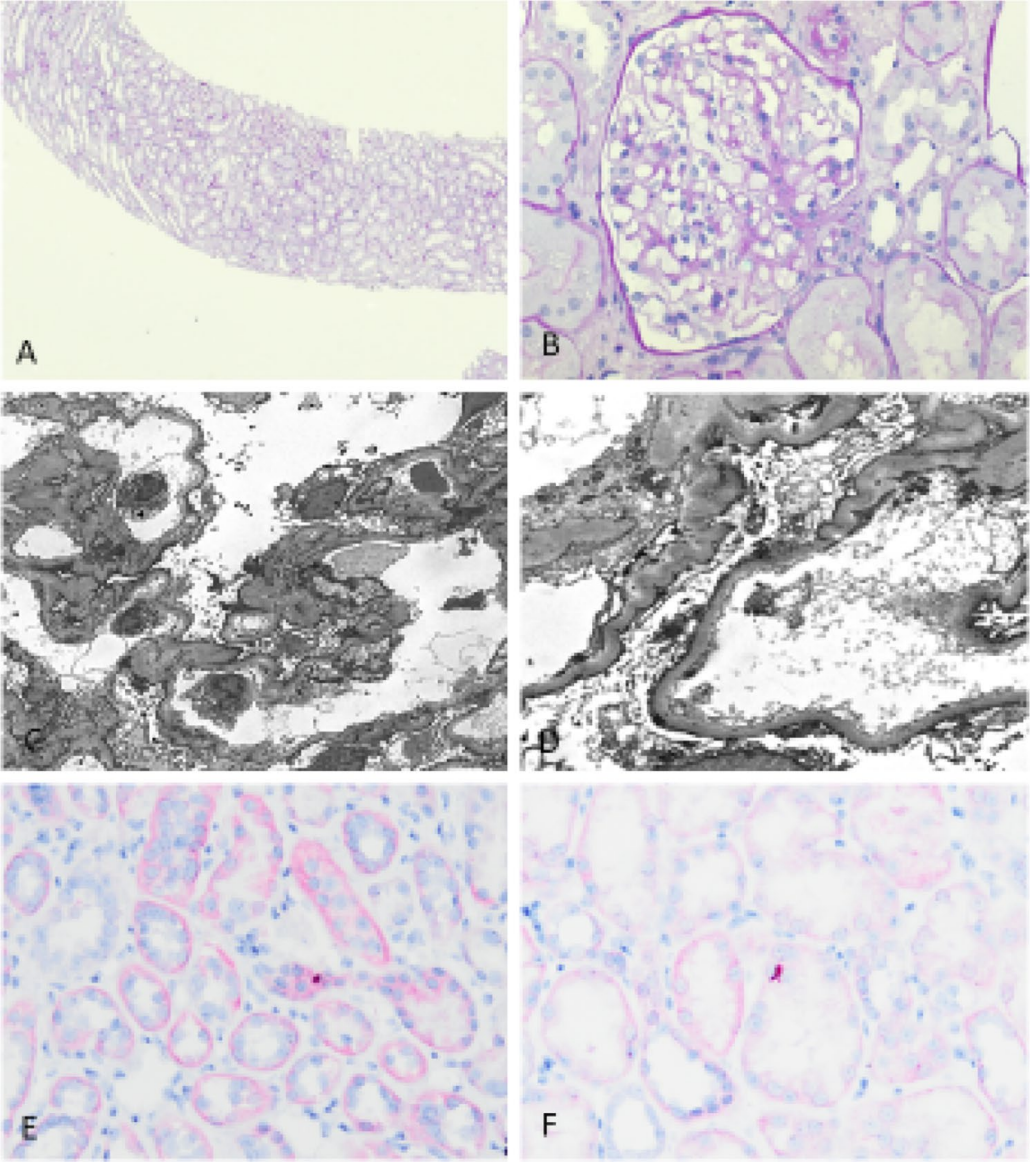
Kidney allograft biopsy showing (**a**, × 20) a quite unremarkable renal biopsy in PAS staining as well as (**b**, × 40) normal glomeruli. (**c**, × 2000, **d** × 5000). Electron biopsy showing diffuse foot process effacement without visible virus particles, and (**e**, **f**, × 20) in situ hybridization using RNA scope visualized SARS-CoV-2 viral RNA as dotted red staining in tubules

**Fig. 2:**
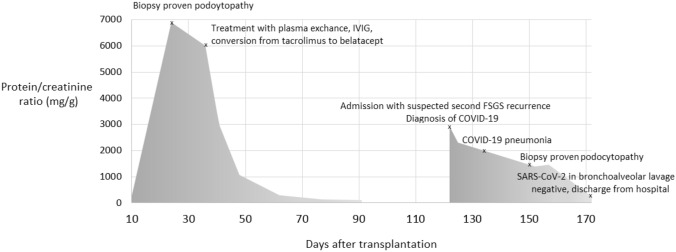
Timeline of proteinuria and clinical course

Suspecting a second recurrence of FSGS, the patient was admitted to hospital for allograft biopsy. At this time, a triage system for SARS-CoV-2 had been established and the patient reported isolated dysgeusia. RT-PCT from the consecutively performed nasopharyngeal swab test was positive for SARS-CoV-2. Kidney biopsy was postponed and the patient was discharged with his standard immunosuppression. At day 27 after the first diagnosis of infection he was admitted to hospital with COVID-19 pneumonia (Supplemental Fig. 1). Proteinuria was 3170 mg/l and albuminuria was 2650 mg/l with stable excretory kidney function (serum creatinine concentration 1.7 mg/dl, eGFR 49 ml/min). Immunosuppression was reduced to hydrocortisone monotherapy (200 mg/days). As symptoms of pneumonia were mild without need for oxygen supplementation, we refrained from administering antiviral drugs. At day 34 of the infection, renal allograft biopsy was performed obtaining 20 glomeruli. PAS staining was rather unremarkable with no signs of cellular or antibody mediated rejection (Fig. 1a, b). Electron microscopy showed marked podocyte effacement (Fig. 1c, d). In situ hybridization (RNA scope 2.5 HD detection kit, Advanced Cell Diagnostic, US) detected positive staining for viral RNA of the SARS-CoV-2 spike protein in tubular epithelial cells (Fig. 2e). Quantification yielded 9 positive signals per biopsy area.

Thus, we hypothesized a SARS-CoV-2-induced recurrence of primary podocytopathy and refrained from plasmapheresis, ivIG, or other intensification of immunosuppression. After recovery from COVID-19 pneumonia, the patient was discharged from hospital.

The first negative nasopharyngeal swab test was obtained seven weeks after the first diagnosis of infection. At this time, nephrotic proteinuria had resolved spontaneously and excretory kidney function corresponded to his baseline values prior to the infection with SARS-CoV-2. Figure 2 presents the course of proteinuria with the corresponding timeline of the clinical course of his infection. The patient provided written informed consent for publication.

## Lessons for the clinical nephrologist

SARS-CoV-2 is characterized by multiorgan tropism including the kidneys. Recent autopsy series indicated that SARS-CoV-2 can infect both tubular and glomerular cells [1, 2]. Whereas tubular cell infiltration may contribute to acute kidney injury, data on a potential clinical correlative to glomerular affection is rare. We describe the first case of nephrotic syndrome in the context of COVID-19 in a renal transplant recipient.

The patient suffered from a recurrence of podocytopathy with FSGS as the underlying disease in his renal transplant. The sudden rise in proteinuria coincided with the first symptoms of COVID-19, spontaneous reconstitution of nephrotic syndrome coincided with recovery from COVID-19. This temporal association strongly argues for a causal relationship. In situ hybridization proved allograft infiltration by SARS-CoV-2. These data demonstrate that the respective post-mortem findings from China and Germany might indeed be of clinical relevance [1]. In the present case SARS-CoV-2 was detected in tubular cells but not in glomeruli. Hence, it may be speculated that besides the potential effects of direct glomerular infection, the inflammatory millieu in COVID-19 may result in an increased production of FSGS causing circulating factors.

The present data expand our understanding of the manifold clinical appearance of COVID-19 [3]. Beyond the respiratory tract the virus exerts clinically relevant effects on vasculature, coagulation, heart, central and peripheral nervous system, intestine, and the kidneys [4, 5]. Moreover, the differential effects of SARS-CoV-2 on tubular and glomerular cells illustrate that even within one organ, the virus can elicit a variety of clinical scenarios. We have previously described the detection of SARS-CoV-2 RNA by in situ hybridization in the interstitium and tubular cells of a renal allograft leading to acute kidney injury [6]. The present case shows that SARS-CoV-2 is able to induce different clinical presentations by infiltration of the same organ. It may be speculated that the expression of ACE-2 is a crucial determinant of organ and compartment involvement in this context.

In terms of his history of primary FSGS the patient provided a predisposition for podocyte injury. It may therefore be questioned whether SARS-CoV-2-associated glomerulopathy is limited to certain risk groups. To the best of our knowledge, only two cases of SARS-CoV-2-associated glomerulopathy have been described so far, both of them in the non-transplant population. Interestingly, these patients were of African origin as well and both of them had collapsing glomerulopathy [7, 8]. African Americans are at increased risk of kidney diseases due to the high prevalence of G1 and G2 as risk alleles of the APOL1 gene. Collapsing glomerulopathy is a typical form of kidney disease in the APOL1 spectrum [9]. These cases illustrate that Africans may be at increased risk for renal disease in COVID-19. Of note, investigation of renal tissue for SARS-CoV-2 RNA by PCR [7] or in situ hybridization [8] remained negative in these cases.

Nephrotic syndrome remitted in the course of COVID-19 during immunosuppressive monotherapy with hydrocortisone. Usually, recurrence of primary FSGS after renal transplant necessitates a substantial increase in immunosuppression—as demonstrated by his first recurrence after transplantation. We refrained from an intensification of immunosuppression during the COVID-19 associated episode for two reasons: First, we did not want to increase the risk of an adverse course of COVID-19. Second, we could not exclude a transient character limited to the time of COVID-19. Thus, the present case shows that immunosuppression can indeed be dispensable in nephrotic syndrome due to COVID-19.

In conclusion, we present a first case of nephrotic syndrome in a renal transplant recipient with COVID-19 and evidence of glomerular affection by the virus. The case broadens the spectrum of renal involvement in COVID-19 from acute tubular injury to glomerulopathy. Thus, it may be wise to test for SARS-CoV-2 prior to initiation of immunosuppression in new onset glomerulopathy during the pandemic.

## Electronic supplementary material

Below is the link to the electronic supplementary material.

Supplementary file1 (DOCX 425 kb)

## Abbreviations

Covid-19: Coronavirus disease 2019
SARS-CoV-2: SARS Coronavirus 2
ACE2: Angiotensin-converting enzyme 2
