# Supplementary material for: Lessons for the clinical nephrologist: recurrence of nephrotic syndrome induced by SARS-CoV-2
Source: J Nephrol. 2020 Sep 5;33(6):1369–72. doi: 10.1007/s40620-020-00855-5 (PMC7474570; doi:10.1007/s40620-020-00855-5)

**Supplement Figure 1:** Thoracic computed tomography showing bilateral ground-glass opacity (arrows) in beginning COVID-19 pneumonia


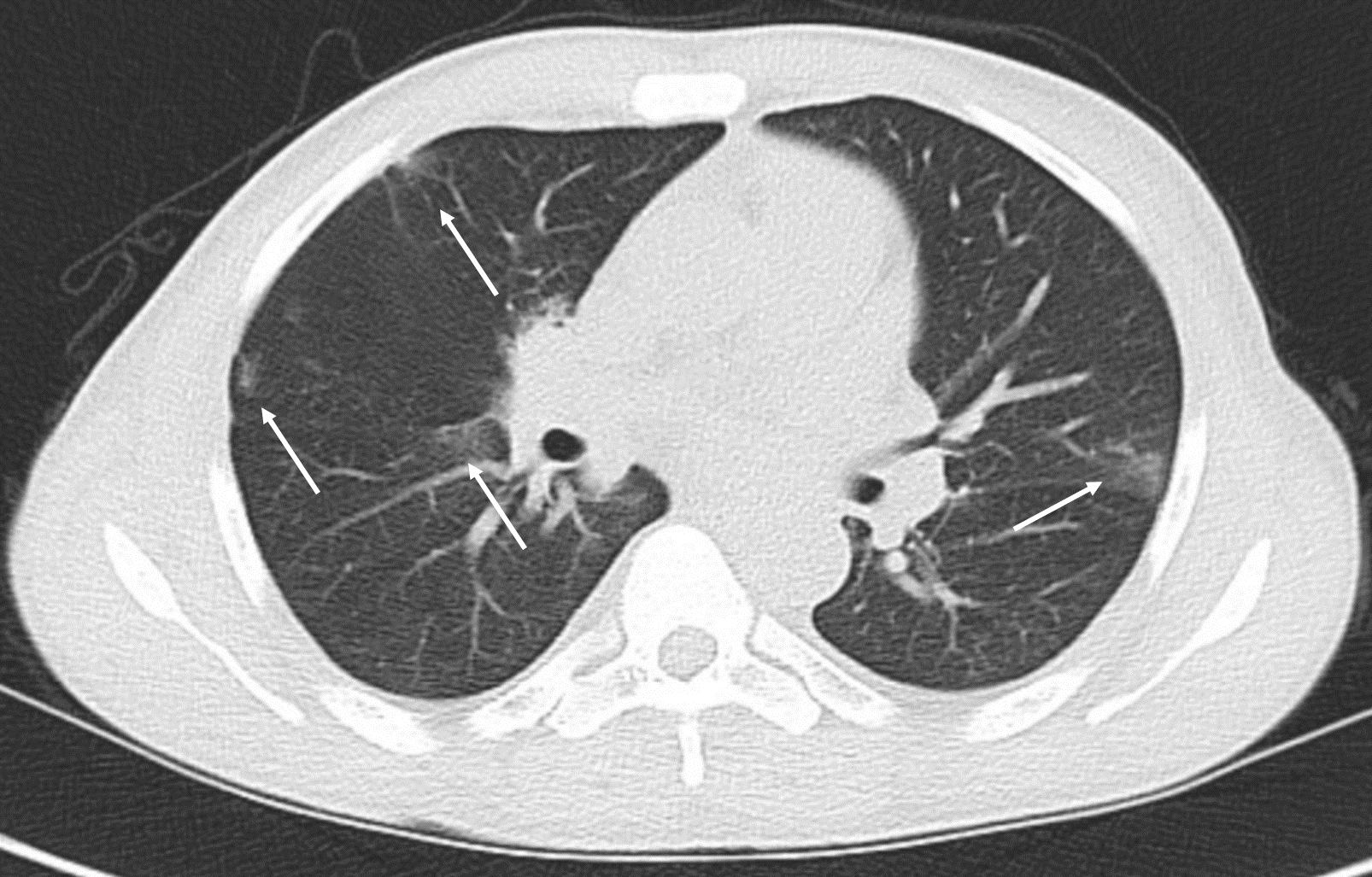

Supplement: Supplementary file 1 — Supplementary file1 (DOCX 425 kb) [file 40620_2020_855_MOESM1_ESM.docx]
